# Psoriatic Arthritis With Dactylitis: A Case Report and Concise Review of Treatment Options

**DOI:** 10.7759/cureus.16966

**Published:** 2021-08-06

**Authors:** Manas Pustake, Tushar Vidhale, Swastik Nadgire

**Affiliations:** 1 Department of Internal Medicine, Grant Government Medical College and Sir JJ Group of Hospitals, Mumbai, IND

**Keywords:** psoriasis, dactylitis, psoriatic arthritis, sausage fingers, therapy

## Abstract

Dactylitis is characterized by generalized swelling of a finger or toe. Though it is commonly seen, the literature on psoriatic dactylitis is scant, with the majority consisting of solitary case reports. The literature on the treatment of dactylitis is considerably more limited. Dactylitis usually responds to non-steroidal anti-inflammatory drugs (NSAIDs) or traditional disease-modifying antirheumatic drugs (DMARDs). Numerous randomized studies have lately shown the effectiveness of different biological agents in the treatment and maintenance of psoriatic arthritis (PsA) and associated dactylitis. In primary care practice, a patient may present with dactylitis without a history of psoriasis. In such cases, an attempt should be made to detect the underlying psoriasis by looking for a psoriatic patch in hidden areas viz. skin folds, groin or scalp. Here, we describe a case of PsA with dactylitis in this case report, with an emphasis on treatment and outcome. We also attempted to focus on the various treatment options for dactylitis.

## Introduction

Psoriatic arthritis (PsA) is inflammatory arthritis caused by psoriasis [1]. Dactylitis has historically been associated with spondyloarthropathies, especially psoriatic arthritis. Dactylitis is characterized by generalized swelling of a finger or toe. It is considered a hallmark clinical feature of PsA [2]. Dactylitis occurs in 16-49% of patients with PsA. It is also seen in cases of tuberculosis, sickle cell disease, sarcoidosis, or syphilis [3,4]. The literature on the treatment of dactylitis is considerably more limited. Dactylitis is unique in terms of pathophysiology, diagnosis, and prognosis since it is associated with radiographic changes in PsA, distinguishing its management from the standard treatment of PsA [5]. The pathophysiology of dactylitis is best described as an initial immune response to biomechanical stress or injury, resulting in inflammation and generalized swelling of the fingers [6]. Despite its prevalence, there are no well-reported cases of psoriatic dactylitis in the literature. In primary care practice, a patient may even present with dactylitis without a history of psoriasis. In such cases, an attempt should be made to detect the underlying psoriasis by looking for a psoriatic patch in hidden areas viz. skin folds, groin or scalp. There are very few drugs with good evidence of benefit in the randomized trials for dactylitis. We are presenting a case of PsA with dactylitis and attempting to explore the literature for evidence of PsA and dactylitis treatments.

## Case presentation

A 35-year-old man presented to us with painful swelling of his right index and ring fingers, as well as the fourth toe on his right foot, which was present for five days. He could not do his daily activities due to severe pain in the affected fingers and toes. His medical history had been unremarkable. His paternal uncle had PsA, which was successfully treated with adalimumab.

**Figure 1:**
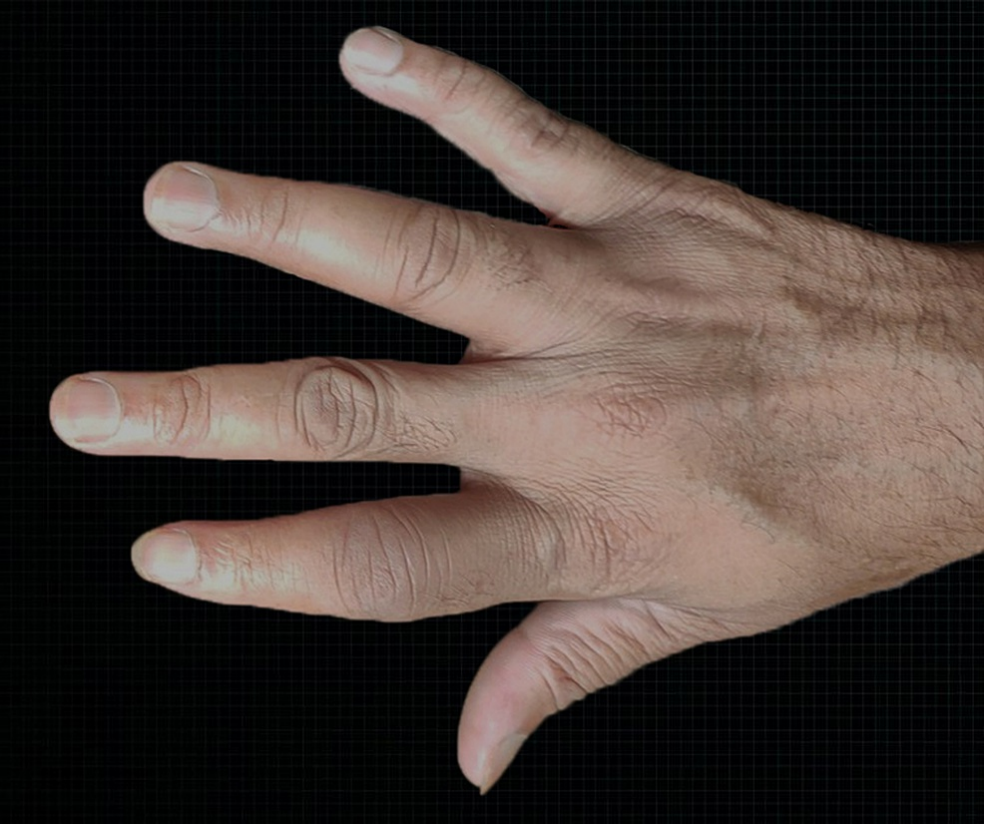
Image showing swelling of right index and ring fingers

**Figure 2:**
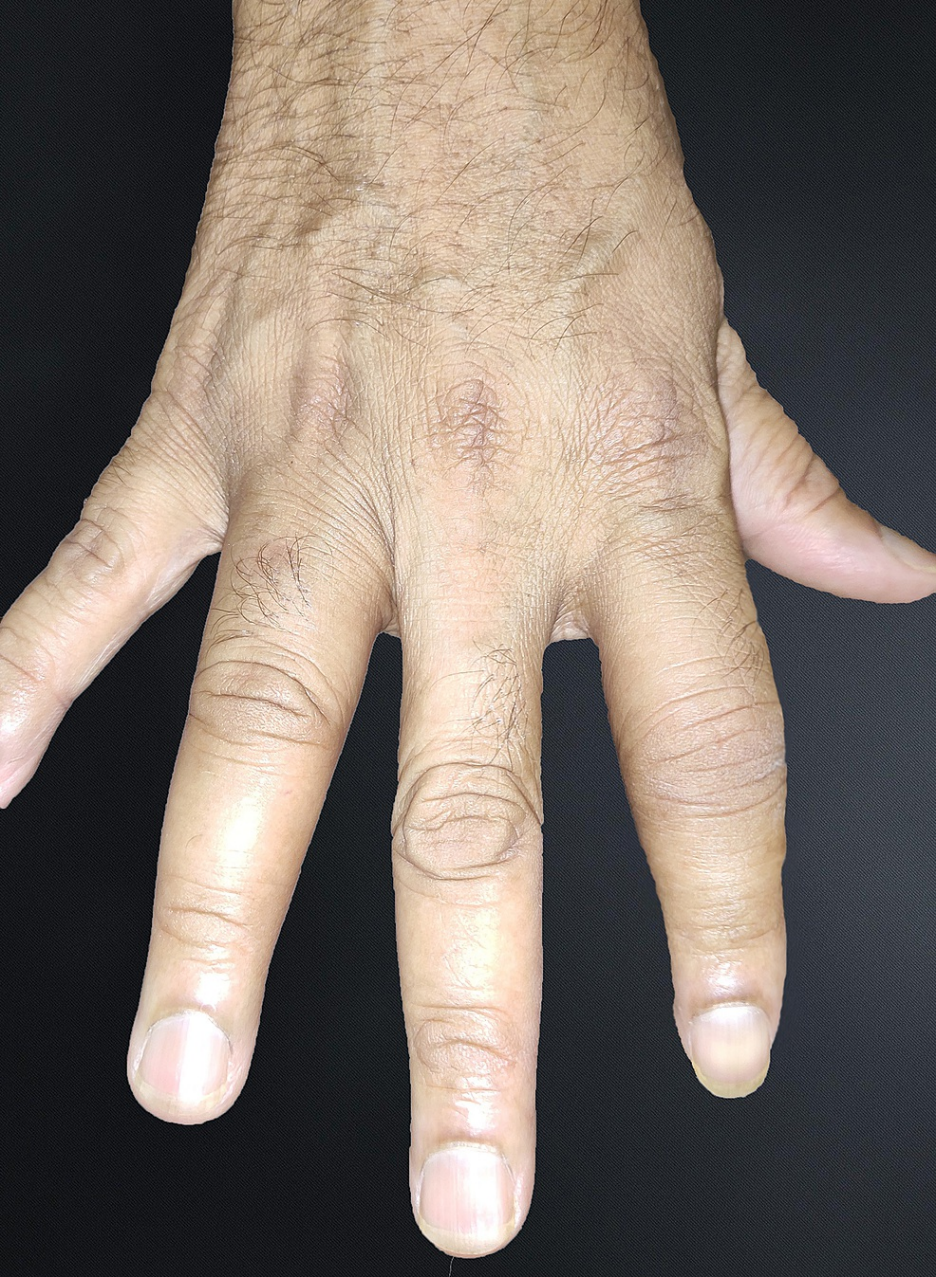
Image showing swelling of right index and ring fingers

**Figure 3:**
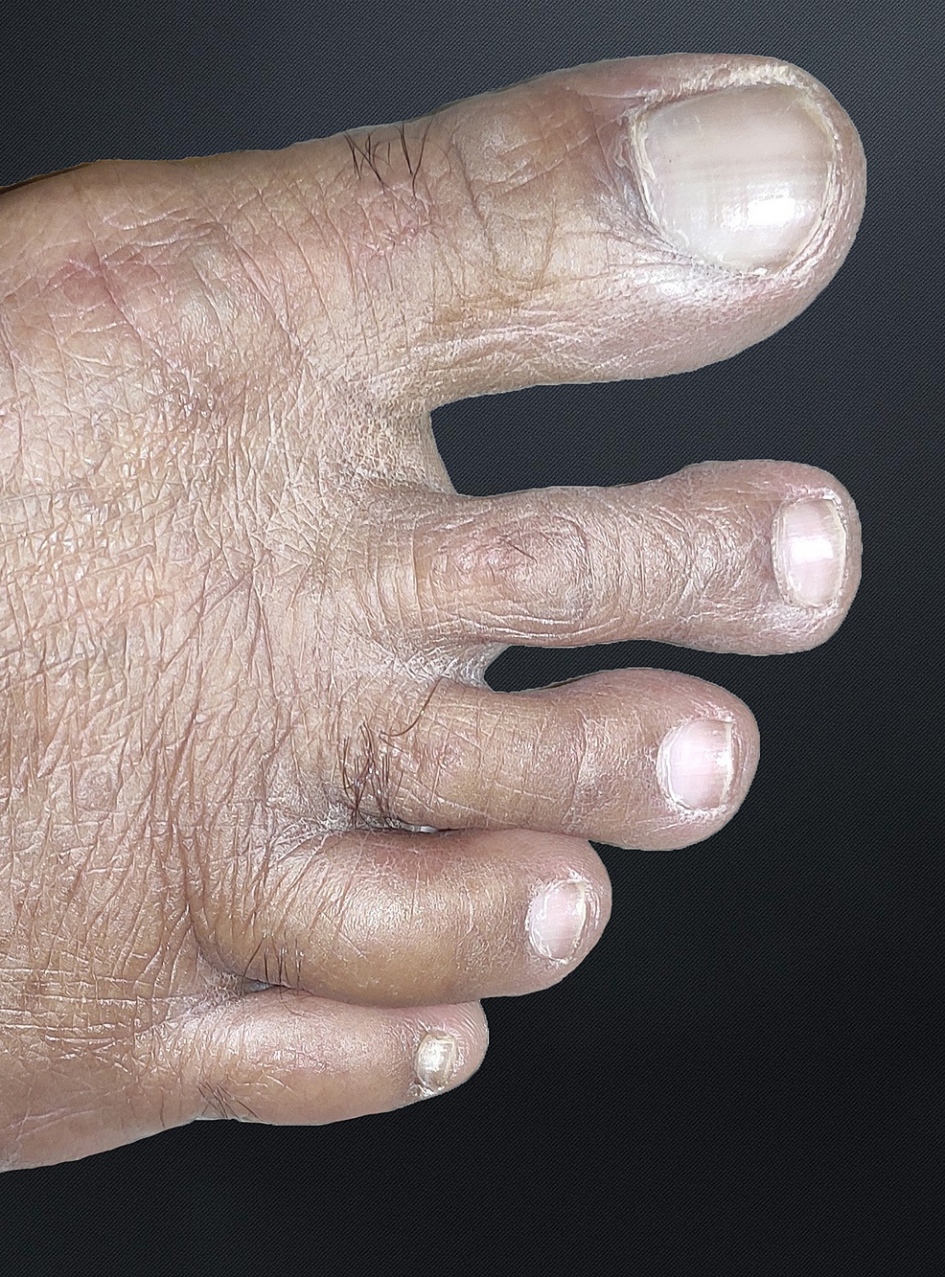
Image showing swelling of the fourth toe of his right foot

**Figure 4:**
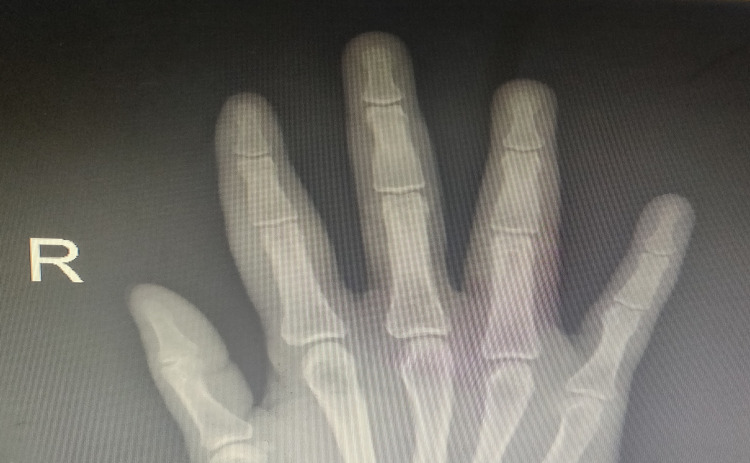
Hand X-Ray depicting mild edema of the soft tissue of the right index and ring fingers

**Figure 5:**
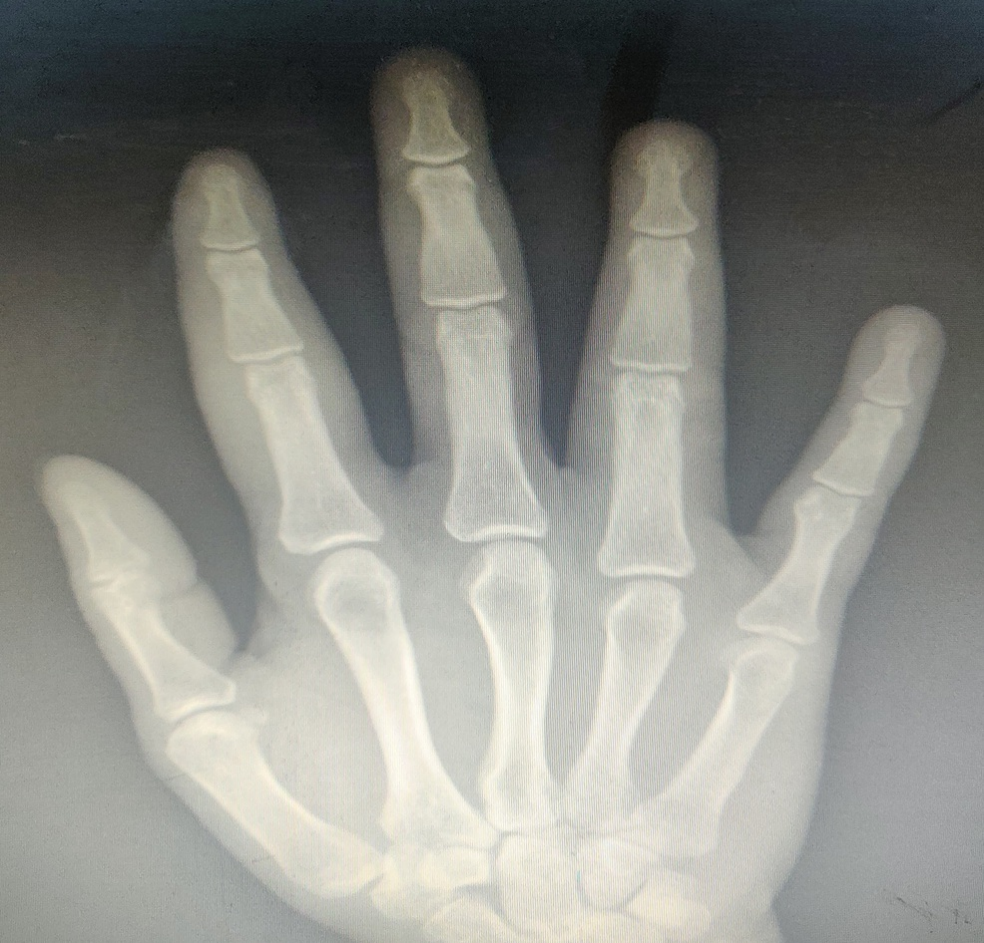
Hand X-Ray depicting mild edema of the soft tissue of the right palm, as well as right index and ring fingers

**Figure 6:**
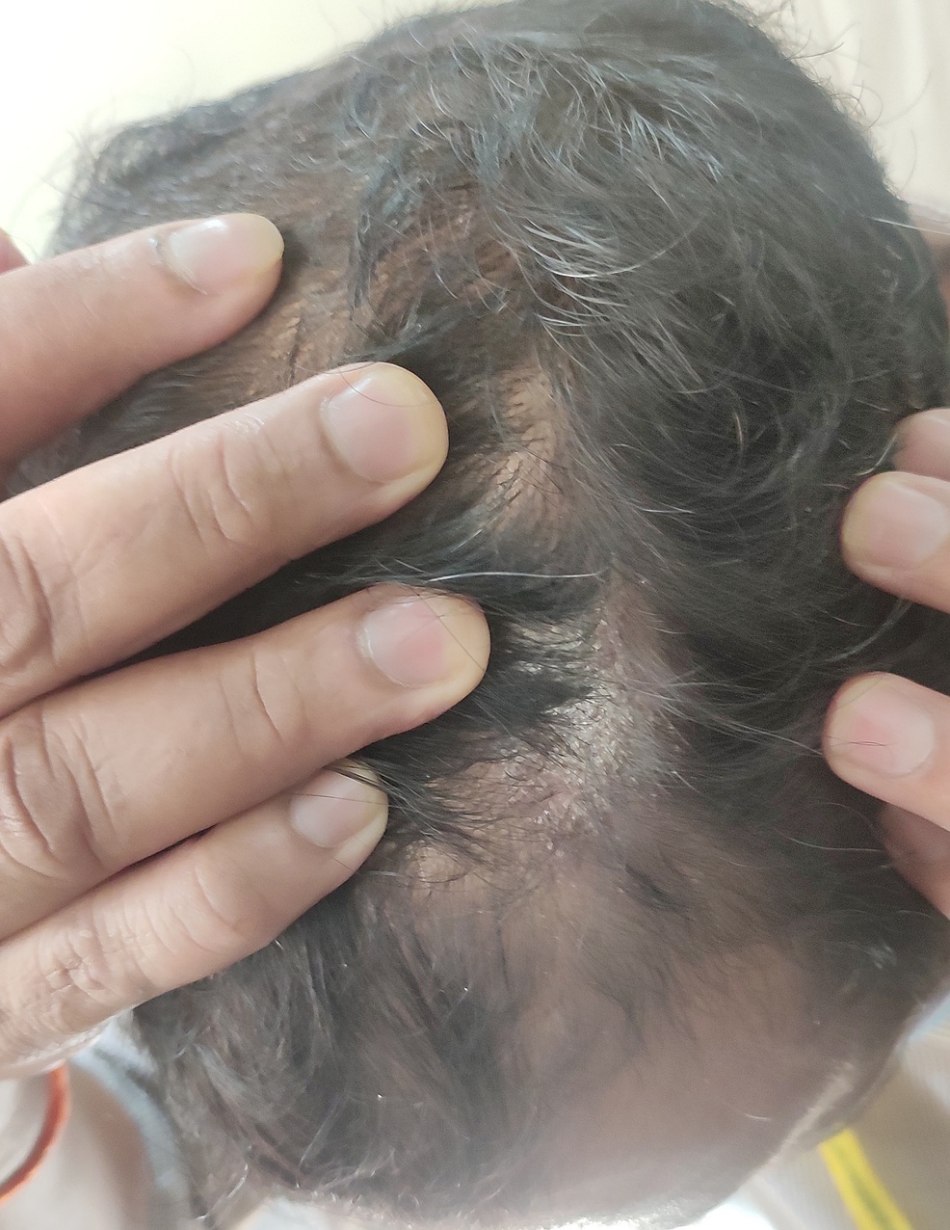
Psoriatic patch with scaling of 3 cm X 2.5 cm was detected on his scalp

Physical assessment revealed tender, fusiform, swollen soft tissues in the affected fingertips, the fourth toe, and swollen palms (Figures 1, 2, 3). Nails were normal in appearance. Hand radiography revealed mild edema of the soft tissue of the index and ring fingers, but no significant joint abnormalities (Figures 4, 5). Dactylitis was diagnosed based on clinical symptoms and radiographic results. Then we attempted to look in the hidden areas such as the scalp, genitals, and skin folds, upon which a psoriatic patch with scaling of 3 cm X 2.5 cm was detected on his scalp (Figure 6). Enthesitis was not present.

Laboratory tests revealed a negative human leukocyte antigen B27 (HLA-B27) test and normal inflammatory markers.

Based on the presence of the psoriatic patch, a negative rheumatoid factor, and dactylitis, we diagnosed him with PsA. He got a total score of 4 on the Classification Criteria for Psoriatic Arthritis (CASPAR), in which a score of 3 or above results in a diagnosis of psoriatic arthritis.

We began the patient on methotrexate 15 mg once a week, folic acid tablets 5 mg every alternate day, and naproxen tablets 500 mg stat, thereafter 250 mg twice a day.

At the two-month and six-month follow-ups, the patient had substantial relief from pain and swelling and was able to continue his daily activities.

## Discussion

Dactylitis is considered a hallmark clinical feature of PsA. It is "a condition in which the metacarpophalangeal joints, as well as the proximal and distal interphalangeal joints, are diffusely swollen to the point that the true joint swelling cannot be identified separately” [3]. Dactylitis occurs in 16-49% of patients with PsA, often early in the disease as the inaugural symptom. Recurrent dactylitis, frequently in the same digit, may be the only manifestation of PsA for months to years [3,4]. PsA usually affects few fingers and/or toes; sometimes simultaneous involvement of most fingers [3]. Our case depicted findings consistent with this.

The pathophysiology of dactylitis is poorly understood. However, it is essentially dependent upon the underlying cause. In the majority of instances, soft tissue is affected along with subcutaneous edema. In PsA, all of these changes may be ascribed to a complex immunological response [7].

When it comes to the treatment of dactylitis, different medications have been used to treat psoriasis with underlying dactylitis, ranging from non-steroidal anti-inflammatory drugs (NSAIDs) to one or more disease-modifying antirheumatic medicines (DMARDs) for the reduction of inflammation in individuals with refractory joint disease [8]. NSAIDs worked great for our patient. There are reports that dactylitis may, however, persist even when treated with NSAIDs [9].

Many physicians suggest injecting corticosteroids into the tenosynovial sheath, joint, or soft tissues since many patients with dactylitis do not respond to NSAIDs [10]. When compared to systemic therapy alone, a local steroid injection into the digital flexor tendon sheath for the treatment of active dactylitis in psoriatic arthritis patients is preferred by some clinicians [11]. But, no evidence showing their effectiveness has been published.

DMARDs were formerly utilized as a therapeutic option for PsA and associated dactylitis. Nonetheless, the Group for Research and Assessment of Psoriasis and Psoriatic Arthritis (GRAPPA) recommends the use of DMARDs, including methotrexate, while the European League Against Rheumatism (EULAR) PsA guidelines do not support methotrexate but do urge starting a biologic therapy immediately [5,8,12].

Biologic medicines have so far been the preferred treatment for PsA and dactylitis. They treat psoriasis as well as synovitis, enthesitis, and non-infectious inflammatory osteitis, which are all common in PsA [13]. Table 1 depicts various clinical trials depicting the efficacy of biologic agents in patients with dactylitis. Several clinical trials have shown significant benefits in dactylitis [14-20]. However, dactylitis was not the main outcome measure in any of these trials.

**Table 1:** Clinical trials depicting the efficacy of biologic agents in patients with dactylitis.

Authors	Trial type	Therapy	Assessment of Dactylitis	Results
Mease et al [15]	Phase II, randomized, placebo-controlled, crossover, double-blind.	Patients were randomized to subcutaneous injections of guselkumab 100 mg or placebo at weeks 0, 4, and every 8 weeks, with a placebo. Crossover to guselkumab at week 24.	Dactylitis was scored on a scale of 0-3 on each digit.	Week 24 through week 56, the mean improvements were greater in the guselkumab group as compared to placebo groups.
Elsa VS et al [16]	Multicentre, randomized, double-blind, placebo-controlled, parallel-design phase 3b trial.	Methotrexate and biologic disease-modifying antirheumatic drugs (bdmards) were randomly assigned to golimumab or placebo, both in combination with Methotrexate.	According to Dactylitis Severity Score (DSS).	By week 24, patients treated with golimumab plus Methotrexate exhibited significantly greater improvements in DSS relative to Methotrexate monotherapy (median change of 5 vs 2 points, respectively; p=0.026).
Mease et al [14]	Multicentre, randomized, double-blind, placebo-controlled, Phase 3 trial.	Randomized 1:1:1 to receive subcutaneous brodalumab 140 mg or 210 mg or placebo at weeks 0, 1, and every 2 weeks up to 24 weeks.	According to Dactylitis Severity Score (DSS).	Significantly higher proportions of patients receiving brodalumab achieved ACR 50/70, dactylitis versus placebo (p<0.01).
Smolen JS et al [17]	Multicentre, randomized, open-label, parallel-group study.	Patients were randomized 1:1 to ixekizumab or adalimumab.	Leeds Dactylitis Index-Basic (LDI-B)	Dactylitis resolution was 83.3% and 81.0% in the case of ixekizumab or adalimumab respectively.
McInnes et al [20]	Phase-3, randomized, double-blind, placebo-controlled, three-arm study	Randomized to receive subcutaneous injections of guselkumab 100 mg every 4 weeks, guselkumab 100 mg at week 0, week 4, and every 8 weeks thereafter, or placebo with crossover to guselkumab 100 mg every 4 weeks at week 24.	-	Guselkumab 100 mg every 4 weeks - 63.5% resolution of dactylitis at week 24 and 74.8% resolution at week 52. Guselkumab 100 mg at week 0, week 4, and every 8 weeks thereafter - 59.4% resolution of dactylitis at week 24 and 75.6% resolution at week 52.
Gladman et al [19]	Randomized, double-blind, placebo-controlled, phase 3 trials.	Patients with psa were randomized to 80-mg ixekizumab every 4 weeks (ixeq4w) or 2 weeks (ixeq2w), after a 160-mg starting dose, or to placebo.	Leeds dactylitis index-basic (ldi-b)	At week 24, ixekizumab-treated patients experienced significantly more resolution than placebo of dactylitis (78% ixeq4w, 65% ixeq2w, 24% placebo)
McInnes et al [18]	Phase 3, randomized, placebo-controlled PSUMMIT 1 and PSUMMIT 2 studies.	Patients were randomized to subcutaneous injections of placebo, ustekinumab 45 mg or ustekinumab 90 mg at weeks 0 and 4 and every 12 weeks. Efficacy was assessed at week 24.	-	Greater proportions of ustekinumab-treated patients had complete resolution of dactylitis at week 24 across the three prior-treatment populations.

Simply put, biologic agents such as ustekinumab, guselkumab, and ixekizumab have been proven to be successful in many clinical studies, indicating that treatment with one of these medications can be started in patients with dactylitis [14-20].

Because dactylitis is a good indicator of PsA severity, its response to therapy may predict PsA prognosis. To the best of our knowledge, no studies have been conducted to predict the therapeutic response to PsA from therapeutic response to dactylitis. There is still a need for further study in this area.

## Conclusions

Dactylitis is the hallmark feature of PsA, the presence of which can highlight the possibility of underlying psoriasis. In general practice, every patient who presents with dactylitis should be checked for psoriatic patches in hidden regions. In this case, we found a psoriatic patch in a patient who had not previously been diagnosed with psoriasis, confirming the diagnosis of PsA. When it comes to the treatment, methotrexate and NSAIDs worked effectively for our patient. We did not need biological agents or steroids to manage the patient. However, so far, biological agents, particularly secukinumab and ustekinumab, are considered as the best available options for the treatment of dactylitis.
